# Comparison of Person-Centered and Cumulative Risk Approaches in Explaining the Relationship Between Adverse Childhood Experiences and Behavioral and Emotional Problems

**DOI:** 10.1177/08862605231153877

**Published:** 2023-02-10

**Authors:** George Hales, Agata Debowska, Richard Rowe, Daniel Boduszek, Liat Levita

**Affiliations:** 1 University of Chester, UK; 2 University of Sheffield, UK; 3 SWPS University of Social Sciences and Humanities, Warszawa, Poland; 4 University of Huddersfield, UK; 5 University of Sussex, Brighton, UK

**Keywords:** child abuse, community violence, mental health and violence, violence exposure

## Abstract

Adverse childhood experiences (ACEs) commonly co-occur, and researchers often estimate their impact using a cumulative risk approach. The person-centered approach offers another approach to operationalize the co-occurrence of ACEs. This study aims to estimate latent classes of ACEs in a sample of U.K. children, examine their relationship with emotional and behavioral problems, and compare the explanatory value of the latent classes to cumulative risk scores. Data were collected among a general population sample of British 10-year-old children extracted from the U.K. Household Longitudinal Study (*N* = 601). Seven items characterized ACEs, comprising parent-report physical discipline, emotional abuse, supervisory neglect, maternal psychological distress, child-report parental educational disinterest, bullying victimization, and adverse neighborhood. Outcome measures were derived from the self-report Strengths and Difficulties Questionnaire including total difficulties, emotional symptoms, conduct problems, hyperactivity, peer problems, and prosocial behavior. Latent class analysis resulted in a three-class solution: low ACEs, household challenges, and community challenges. Compared to the other classes, the community challenges class scored substantially worse on total difficulties, emotional symptoms, and peer subscales. The cumulative risk score was associated with all outcomes except prosocial behavior. Cumulative risk models accounted for a larger proportion of variance compared with the latent class models, except for peer problems which the person-centered model explained better. This study confirms that ACEs are associated with impairment in child functioning, and that both person-centered and cumulative risk approaches can capture this relationship well. Specifically, the person-centered approach demonstrated how co-occurring risk factors in the community challenges class produced particularly poor internalizing outcomes.

## Introduction

Exposure to multiple adverse childhood experiences (ACEs) has been linked to a multitude of negative sequelae. However, as adversities do not confer determined outcomes, causal pathways are likely to feature indirect mechanisms. One potential way to elucidate potential indirect mechanisms is to explore how ACE risks cluster together. The present study evaluates two alternative operationalizations of multiple ACEs.

Several studies examined the dose–response relationship between adversities and outcomes including alcoholism, drug abuse, depression, suicide attempts, and smoking (e.g., Edwards et al., 2003; Felitti et al., 1998). More recently, Hughes et al.’s (2017) meta-analysis of 137 studies found exposure to four or more ACEs (compared to no ACEs) substantially worsened outcomes, with particularly strong risks associated with problematic drug or alcohol use, self-directed or interpersonal violence, sexual risk-taking, and mental illness. Given that 23% of children in the United Kingdom (Hughes et al., 2021) and 35% of children in North America (Bellis et al., 2019) experience multiple ACEs, the findings presented above indicate a concern for public health.

The predominant approach in operationalizing ACEs is the cumulative risk approach, which assesses the dose–response effect of ACEs. This approach treats each categorical ACE as equally additive to a general effect, which provides insight for general risk, but provides limited insight into the risks of exposure to specific ACE clusters. Indeed, this highlights a problem for intervention strategies because the needs of children with high ACE scores could vary widely, meaning interventions cannot be tailored (see Finkelhor, 2018, for a discussion). Alternatively, the study of co-occurring patterns of ACEs might be beneficial for the development of specific screening tools and patient-centered interventions.

One such method is the person-centered approach, which uses latent class analysis (LCA) with categorical data or latent profile analysis with continuous data to identify unobserved groups defined by patterns of co-occurring items (Lanza & Rhoades, 2013). A key assumption of this approach is that the distribution of ACEs can be explained by groups of individuals who have experienced similar patterns of ACEs. Each group has an estimated likelihood of the presence of each item. Classes can be distinguished quantitatively (i.e., high/low probability of all items) and qualitatively (i.e., high probability of some items, low probability of other items). Membership of computed latent classes can be used to estimate outcomes associated with that class, or to highlight groups at higher risk of class membership (e.g., Debowska et al., 2018). The effects of different combinations of ACEs can be ascertained through the person-centered approach, which might be informative for screening and intervention strategies.

LCA applied to child maltreatment has resulted in some population-level trends. For instance, a systematic review of child maltreatment LCA studies found that a three- or four-class solution is fairly typical, quantitatively distinct classes (i.e., no/low abuse and poly-victimization) were common, and while qualitative classes varied between studies a sexual abuse class was observed consistently (Debowska et al., 2017). Studies varied in using child, adolescent, and adult samples, and used a range of data collection methods such as self-report, parent-report, and child welfare records, all of which may have contributed to variation in class solutions.

### Formal Comparisons Between Cumulative Risk and Person-Centered Approaches

Studies utilizing both cumulative risk and person-centered approaches agree that greater numbers of ACEs are associated with worse outcomes, although some LCA studies have demonstrated that qualitative classes are also informative. One study using a community sample of children identified a seven-class model (Lanier et al., 2018), where the classes with the strongest association to poor health outcomes were a high ACEs class, and a class with high probability of parental mental illness and poverty. Another study sampled undergraduate students and found a four-class model comprising high ACEs, moderate risk of nonviolent household dysfunction, emotional and physical abuse, and low ACEs (Merians et al., 2019). While the high ACEs group was associated with the most severe outcomes, the emotional and physical abuse class only differed slightly from the high ACEs class, which implies that this qualitative class is particularly potent.

Formal comparisons of the explanatory utility approaches have so far produced inconclusive results. For instance, Merians et al. (2019) compared approaches using 9 ACE items among a sample of undergraduate students, with the outcomes concerning mental health, physical health, alcohol use, and academic performance. A four-class solution was compared to nominal groupings of 0, 1, 2, 3, 4, and 5 or more ACEs; both models explained similar magnitudes of variance. Another study compared LCA and cumulative risk approaches in relation to chronic inflammation outcomes (Lacey et al., 2020). This study found a four-class solution (low ACEs, polyadversity, parental mental illness and substance misuse, maltreatment and conflict). While the cumulative risk approach produced a dose–response relationship for three inflammation markers, the person-centered approach produced different outcomes for each class. The polyadversity and maltreatment and conflict classes were associated with the highest scores for different inflammation markers. This study presents subtle differences in outcomes by latent class typology, and suggests that the combination of maltreatment and familial conflict poses a specific risk for chronic inflammation, which was not captured by the cumulative risk approach.

There are several limitations in the literature which could contribute to inconsistent findings. First, much research relies on retrospective data. A recent meta-analysis found poor agreement between prospective and retrospective measures of child maltreatment (Cohen’s *k* = 0.19), so study design could impact results (Baldwin et al., 2019). The reliance on adult retrospective data limits understanding of latent classes in children due to substantive biases of memory, so prospective data should be preferred. Second, many studies utilize samples with a wide age range (e.g., Lanier et al., 2018). This can compromise validity because participants aged 11 to 18 years have had more time to accumulate adversity than 10-year-olds; ACEs may have different impacts at different ages. Third, many studies use American samples (e.g., Lanier et al., 2018; Merians et al., 2019), which might limit the generalizability to other populations. Fourth, there is inconsistency in how ACEs are conceptualized. The original specification of ACEs included seven items (Felitti et al., 1998), but the number of adversities included in measurements varies (Hales et al., 2022). A recent study recommended the inclusion of bullying victimization, community violence exposure, and social ostracism due to the strengthened predictive power of risk scores that included these items (Finkelhor et al., 2015).

### The Current Study

The current study aims to (a) explore latent classes of ACEs (physical discipline, emotional abuse, supervisory neglect, parental educational disinterest, maternal psychological distress, bullying victimization, adverse neighborhood) in a U.K. household sample of 10-year-old British children; (b) identify relationships between identified classes and child behavior and emotional problems; and (c) compare latent class and cumulative risk approaches in explanatory validity of child behavior and emotional problems. This current study will address several limitations in the exploration of cumulative risk and person-centered approaches to ACEs. First, ACEs were measured concurrently using child- and parent-report data, so the present study does not rely on retrospective self-report data. Second, the sample was restricted to children aged 10 years old which eschews the confounding effects of age. Third, the population sampled was a non-American community sample, which supplements the evidence base currently reliant on American samples. Fourth, the ACEs included were chosen to reflect the broadening concept of ACEs, which resulted in the inclusion of ACEs such as bullying victimization, adverse neighborhood (to approximate community risks), and (parental) educational disinterest (to approximate a milder form of educational neglect). This study will contribute to the growing knowledge of how ACEs co-occur, and the explanatory value of person-centered and cumulative risk approaches in operationalizing ACEs.

## Method

### Sample and Data

We used prospectively collected data from the general population youth sample at wave 3 of the U.K. Household Longitudinal Study (UKHLS) to perform cross-sectional analysis. This wave of data was collected during 2011 to 2013, from approximately 24,000 households (University of Essex, 2020). This wave was chosen because it is the earliest wave that included the variables of interest, which minimized the effect of attrition. Only data concerning children aged 10 years old were used. Data were collected through self-completed and parent-reported questionnaires. Parent-report data concerns variables related to parents (i.e., discipline, stress). Oral consent was given by participants at each wave. For participation, adults received £10 vouchers and children received £3 vouchers. The University of Essex Ethics Committee approved data collection. Data were accessed after End User License approval from the UK Data Service (https://beta.ukdataservice.ac.uk/datacatalogue/studies/study?id=6614).

Only observations with complete ACEs data were included in analysis, which resulted in *n* = 119 observations being dropped. The final sample used for analysis was *N* = 601, with a balanced sample of male (48.8%, *n* = 293) and female (51.2%, *n* = 308) children. Most of the samples were White British or Irish (82.5%) and the remaining 17.5% were Asian, Black, or from mixed ethnic backgrounds, which closely represents the U.K. population (ONS, 2012). Regarding the social and family context of the sample, most lived in urban areas (76.2%), a small minority of mothers obtained no formal qualifications (9.8%), a minority were living in relative poverty (25.3%), and a fair proportion had a stepparent living with them (31.6%). Regarding the size of the families, a minority of the sample came from single child families (18.8%), most had one sibling (48.4%), some had two siblings (22%), and the remainder had three or more siblings (10.8%).

#### Measures

Confidential computer-assisted self-report data from child participants and parent-report data were retrieved to create variables representing ACEs. In total, seven binary adversity indicators were created, see Supplemental Table 1 for all the contributing items.

#### Parent-Report Adversities

Three parent-reported adversities, *physical discipline* (five items, e.g., “I use physical punishment as a way of disciplining [child’s name]”), *emotional abuse* (two items, e.g., “I scold and criticise to make [child’s name] improve”), and *supervisory neglect* (one item, “I punish [child’s name] by putting him/her somewhere alone with little or no explanation”), were adapted from the parent-report parenting styles questionnaire (see Robinson et al., 1995). All item responses followed a 5-point Likert scale: “never,” “once in a while,” “about half the time,” “very often,” and “always.” Items were dichotomized for LCA, defined as present for: “about half the time,” “very often,” or “always,” and absent for “never” or “once in a while.” “Once in a while” was treated as absent to follow the approach taken elsewhere (see Felitti et al., 1998) where psychological and physical abuse were only recorded as present if parents “often or very often” engaged in a behavior.

*Maternal psychological distress* was self-reported by mothers using the Short General Health Questionnaire (GHQ-12; Goldberg & Williams, 1988). The GHQ is designed to screen for psychiatric disorders via a self-administered scale. The summed caseness scale gives values between 0 (least distressed) and 12 (most distressed), one example item being, “Have you recently been feeling unhappy and depressed?”. Researchers have previously found that using 3 as a cutoff provides a good balance of sensitivity and specificity for screening mental illness diagnoses (Goldberg et al., 1998). For analysis, maternal psychological distress was dichotomized so that values between 3 and 12 were coded as present, values between 0 and 2 were coded as absent.

#### Child Self-Report Adversities

Items adapted from child self-report questionnaires were dichotomized from 5-point Likert scales although the response items differ slightly. We identified three ACEs: *educational disinterest* (two items, e.g., “My parents are interested in how I do at school”), *bullying victimization* (two items, e.g., “How often do you get physically bullied at school?”), and *adverse neighborhood* (two items, e.g., “How safe would you feel walking alone in this area after dark?”). For educational disinterest, responses of “hardly ever” or “never” were coded as present, and “always” or “nearly always,” “sometimes,” and “not sure” as absent; for bullying victimization “a lot” or “quite a lot” were coded as present, and “not much” or “never” as absent. Adverse neighborhood had two items for which the responses differed: “a bit unsafe” and “very unsafe” were coded as present, and “very safe” or “fairly safe” as absent for the question about safety; and “a bit of a worry” and “a big worry” coded as present, and “an occasional doubt” and “not a worry at all” as absent for the question about worrying about being a victim of crime.

#### Strengths and Difficulties Questionnaire

The self-report Strengths and Difficulties Questionnaire (SDQ) comprises five subscales each containing five items. The subscales measure “emotional symptoms” (e.g., “I am often unhappy, depressed or tearful”), “conduct problems” (e.g., “I get very angry and often lose my temper”), “hyperactivity” (e.g., “I am restless, I cannot stay still for long”), “peer problems” (e.g., “I would rather be alone than with people of my age”), and “prosocial behaviors” (e.g., “I try to be nice to other people. I care about their feelings”). Each item is scored from 0 to 2 as “not true,” “somewhat true,” or “certainly true,” making the total score for each subscale between 0 and 10. SDQ scores were derived in the UKHLS dataset prior to researchers gaining access, only the derived scale variables were retrieved from the dataset. Subscale scores were marked as missing if more than two out of five items are missing. However, if only one contributing item was missing a response, the subscale score was retained. The total difficulties score (0–40) was a sum of the emotional symptoms, conduct problems, hyperactivity, and peer problems scales. The SDQ is useful in screening for psychiatric problems in children (R. Goodman et al., 2000) and it shows good predictive validity (A. Goodman & Goodman, 2009). Internal consistency for each scale was estimated using Cronbach’s alpha: total difficulties (α = .81), emotional problems (α = .64), peer problems (α = .56), conduct problems (α = .58), hyperactivity (α = .64), and prosocial behaviors (α = .69). Others have recommended caution in interpreting results regarding conduct problems and peer problems (see Sharratt et al., 2018).

### Data Analysis

LCA was utilized to explore the number and nature of qualitatively homogeneous patterns of ACE exposure (physical discipline, emotional abuse, supervisory neglect, maternal psychological distress, parental educational disinterest, bullying victimization, and adverse neighborhood). Models of between 2 and 7 classes were specified and compared via fit indices. No single index distinguishes the best model. We tested relative model fit by comparing *k* class models to *k* − 1 class models, using conventional indices such as Akaike Information Criteria (AIC; Akaike, 1974), Bayesian Information Criteria (BIC; Schwarz, 1978), sample-size-adjusted BIC (SSABIC; Sclove, 1987), the Lo–Mendell–Rubin adjusted likelihood test (LMR-LRT; Lo et al., 2001), parametric bootstrapped likelihood ratio test (BLRT; Arminger et al., 1999), and entropy values (Ramaswamy et al., 1993). The AIC, BIC, and SSABIC are used similarly; lower values for model with *k* number of classes compared to *k* − 1 indicate a model with better relative fit. LMR-LRT and BLRT test relative fitness through a significance test by comparing model *k* to *k* − 1. Larger entropy values indicate a larger proportion of correctly classified observations, where values approaching 1 indicate better classification of observations. Simulation studies found that the BLRT test performed best, followed by the BIC and SSABIC values (see Nylund et al., 2007), and that SSABIC improves on BIC when sample size is less than 1,000 (Yang, 2006). For each model the AIC, BIC, SSABIC, LMR-LRT, BLRT, and entropy values are presented. As our sample size is relatively small, greater emphasis is placed on SSABIC than on AIC and BIC, but the model with best fit should have high agreement between AIC, BIC, and SSABIC, and the LMR-LRT and BLRT significance tests. Entropy values will be used to judge whether the model solution categorizes observations to an acceptable level (>.80; Ramaswamy et al., 1993).

To explore relationships between most likely class membership and child behavior and emotional symptoms, ANOVAs were run with latent class membership as the predictor variable, and SDQ scales (total difficulties, emotional symptoms, conduct problems, hyperactivity, peer problems, prosocial behavior) as the outcomes. Cohen’s *d* values were estimated to compare the effect of belonging to each class. ANOVAs were repeated using the cumulative risk score (summed dummy indicators of exposure to adversity) with the same number of groups as the latent class groupings. All ANOVAs were repeated with sex and ethnicity included as covariates. Direct comparisons between person-centered and cumulative risk models were made by computing Hay’s omega-squared (ω^2^) for both sets of models. Regressions were computed using dummy coded latent class and cumulative risk groupings in the model. LCAs were conducted using Mplus Version 8.6 (Muthén & Muthén, 1998–2017), while data management and other analyses were conducted using Stata/MP 16 (StataCorp, 2019).

## Results

### Descriptive Information

Table 1 shows the least frequent ACE was supervisory neglect (3.5%), and the most frequent was adverse neighborhood (34.6%). The average number of ACEs reported was 1.29 (*SD* *=* 1.11) (range 0–6). Most participants reported at least one ACE (74.9%) but only 4.2% reported four or more ACEs. It is worth noting that the mean ACEs score for males (*M* = 1.42, *SD* = 1.33) seemed higher than that for females (*M* = 1.18, *SD* = 1.08), and the ACEs score seemed higher for ethnic minorities (*M* = 1.66, *SD* = 1.32) than that for White (*M* = 1.22, *SD* = 1.04) participants.

**Table 1.** Observed Proportions of Adverse Childhood Experiences in Whole Sample and by Sex and Ethnicity.

Adversity	Whole Sample	Male	Female	White	Ethnic Minority
Physical discipline (%)	6.5	8.2	4.9	5	13.3
Emotional abuse (%)	30.6	35.8	25.7	28.4	41
Supervisory neglect (%)	3.5	4.4	2.6	2.4	8.6
Maternal psychological distress (%)	26.3	28.7	24	24.6	34.3
Educational disinterest (%)	12.8	14.7	11	12.1	16.2
Bullying victimization (%)	15.1	19.8	10.7	15.9	11.4
Adverse neighborhood (%)	34.6	30	39	33.3	41
ACEs score mean (*SD*)	1.29 (1.11)	1.42 (1.13)	1.18 (1.08)	1.22 (1.04)	1.66 (1.32)

*Note.* Whole sample, *N* = 601; male, *n* = 293; female, *n* = 308; White, *n* = 496; ethnic minority, *n* = 105. ACEs = adverse childhood experiences.

### Person-Centered Approach

**Figure 1.**
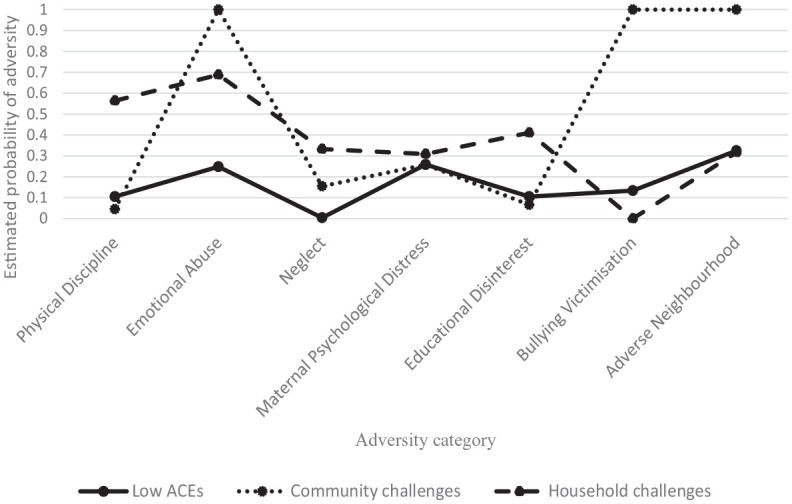
Model-estimated class-specific item-probability profile plot of three-class model.

Table 2 shows enumeration statistics for models specifying 2 to 7 latent classes. AIC and SSABIC values as well as BLRT significance test favored the three-class model, whereas BIC values favored the two-class model. Entropy values for models of 3 to 7 classes indicated good classification of observations; relative fit statistics for models of 4 to 7 were unfavorable. The three-class solution was selected for further analysis. Item endorsement probabilities for each class are presented graphically in Figure 1.

**Table 2.** Class Enumeration Statistics for Latent Class Models of 2 to 6 Classes of Adverse Childhood Experiences.

No. of Classes	Log-Likelihood	AIC	BIC	SSABIC	Entropy	LRT adjusted (*p*)	BLRT
2	−1,797.12	3,524.24	**3,690.219**	3,642.598	.736	**.003**	**<.001**
3	−1,780.624	**3,607.249**	3,708.416	**3,635.397**	**.876**	**.013**	**<.001**
4	−1,773.031	3,608.062	3,744.418	3,646.002	**.903**	**.047**	.2
5	−1,767.245	3,612.49	3,784.035	3,660.221	**.84**	.654	.6
6	−1,762.265	3,618.531	3,825.265	3,676.052	**.855**	**.022**	1
7	−1,758.517	3,627.035	3,868.958	3,694.347	**.863**	.285	.6

*Note*. Boldface indicates acceptable values for each criterion (entropy is evaluated by a cutoff of .8; LRT adjusted and BLRT by an alpha value of .05, while AIC, BIC, and SSABIC are evaluated by comparison with *k* − 1 models). ACEs = adverse childhood experiences; BIC = Bayesian Information Criteria; SSABIC = sample-size-adjusted BIC.

Class 1 comprised the majority of the sample (*n* = 540, 89.9%) and was labeled “*low ACEs*” due to low endorsement probability of all items. Class 2 comprised a minority of the sample (*n* = 36, 6%) and was labeled “*household challenges*” due to the moderate to high probabilities of emotional abuse and physical discipline. Remaining items were of low probability. Class 3 also comprised a minority of the sample (*n* = 25, 4.2%) and was labeled “*community challenges.*” This class was characterized by high probabilities of bullying, adverse neighborhood, and emotional abuse. Other items were of low probability.

Six one-way ANOVAs were computed using latent class groupings as the independent variables and the SDQ scales as outcome variables (see Table 3). For total difficulties, emotional symptoms, and peer problems, significant *F* values were observed (after Bonferroni correction). Additionally, ANOVAs repeated with sex and ethnicity included as covariates remained significant for total difficulties, emotional symptoms, and peer problems (see Supplemental Table 2). Group comparisons were made through observation of the means and standardized effect sizes (Cohen’s *d*). Effects of .2, .5, and .8 were treated as small, medium, and large, respectively (Cohen, 1992).

**Table 3.** Person-Centered Models and Comparison of Strengths and Difficulties Subscale Outcomes Between Identified Latent Classes.

Outcome	Outcome Means per Class (*SD*)			Cohen’s *d* [95% Confidence Interval]			*F*	*p*	ω^2^
	Low ACEs	Household Challenges	Community Challenges	Community Challenges vs. Low ACEs	Household Challenges vs. Low ACEs	Community Challenges vs. Household Challenges			
Total difficulties	9.91 (5.33)	10.29 (5)	15.28 (5.71)	**1.00 [0.60, 1.41]**	0.07 [−0.27, 0.41]	**0**.**94 [0.40, 1.48]**	12.16	<.001	.036
Emotional problems	2.54 (2.04)	2.75 (1.95)	4.12 (2.11)	**0.77 [0.37, 1.17]**	0.1 [−0.24, 0.44]	**0.68 [0.15, 1.2]**	7.22	<.001	.02
Conduct problems	1.98 (1.57)	2.44 (1.80)	2.76 (1.61)	**0.50 [0.10, 0.90]**	0.30 [−0.04, 0.63]	0.18 [−0.33, 0.69]	4.18	.016	.011
Hyperactivity	3.65 (2.15)	3.81 (2)	4.56 (1.80)	**0.43 [0.02, 0.83]**	0.07 [−0.27, 0.41]	0.39 [−0.12, 0.91]	2.22	.109	.004
Peer relationship problems	1.72 (1.64)	1.46 (1.56)	3.84 (1.77)	**1.29 [0.88, 1.70]**	−0.16 [−0.51, 0.18]	**1.44 [0.86, 2.01]**	20.71	<.001	.062
Prosocial behavior	8.35 (1.61)	8.14 (1.76)	7.92 (1.80)	−0.27 [−0.67, 0.13]	−0.13 [−0.47, 0.21]	−0.12 [−0.63, 0.39]	1.09	.337	.0003

*Note*. *Low ACEs*, class *n* = 540; *Household challenges*, class *n* = 36; *Community challenges and emotional abuse*, class *n* = 25. Sample size for each model varies between 595 and 598 dependingon the occasional missing data. Extreme values were winsorized to the lower/upper extreme values. Boldface indicates significant group differences where confidence interval does not cross 0. Bonferroni corrected alpha, α = .003.

The *community challenges* class had the highest score for each SDQ scale (excluding prosocial behavior) compared to the *low ACEs* classes. Compared to the *household challenges* class, the *community challenges* class had a higher total difficulties, emotional problems, and peer problems. Effect sizes indicated large differences between the *community challenges* class to the *low ACEs* class for total difficulties and peer problems, and medium differences for emotional symptoms and conduct problems, all with the *community challenges* class scoring higher. There were also large differences between *community challenges* and *household challenges* classes for total difficulties and peer problems, and moderate differences in magnitude for emotional symptoms, again all with the *community challenges* class scoring higher. Differences between *household challenges* and *low ACEs* classes were nonsignificant based on effect size confidence intervals.

### Cumulative Risk Approach

Table 3 presents the cumulative risk approach in assessing the relationship between adversities and SDQ scales. Groups were created to reflect the same number of groups as latent classes (0–1 ACEs, 2–3 ACEs, 4 or more ACEs). ANOVAs indicated that the cumulative risk grouping of adversities was significantly associated with all SDQ scales except prosocial behavior. All ANOVAs were rerun with sex and ethnicity included in the model, which did not substantively alter the relationships (see Supplemental Table 3). Comparisons between the 4 or more ACEs group and 0 to 1 ACEs produced the largest effect sizes, specifically large for emotional symptoms and total difficulties, and moderate for peer problems and conduct problems. Comparisons between 2–3 ACEs and 0–1 ACEs showed small differences for emotional problems, conduct problems, hyperactivity, and peer problems and a moderate difference for total difficulties.

### Comparison Between Person-Centered and Cumulative Risk Models

Comparisons between person-centered and cumulative risk approaches were made using Hay’s ω^2^, presented in both Tables 3 and 4. Statisticians have identified values of .01, .06, and .14 as estimates of small, medium, and large magnitudes, respectively (Kirk, 1996). For total difficulties, emotional symptoms, conduct problems, and hyperactivity, the cumulative risk models accounted for more variance. For peer problems, the latent class model accounted for more variance. Both latent class and cumulative risk models accounted for small or medium magnitudes of variance for total difficulties, emotional symptoms, conduct problems, hyperactivity, and peer problems.

**Table 4.** Comparison of Strengths and Difficulties Subscale Outcomes Among Cumulative Risk Groupings.

Outcome	Outcome means per class (*SD*)			Cohen’s *d* [95% Confidence Interval]			*F*	*p*	ω^2^
	0–1 ACEs	2–3 ACEs	4 or more ACEs	4 or more vs. 0–1	2–3 vs. 0–1	4 or more vs. 2–3			
Total difficulties	9.15 (5)	11.96 (5.72)	13.08 (5.46)	**0.78 [0.37, 1.20]**	**0.54 [0.36, 0.72]**	0.20 [−0.23, 0.62]	21.62	<.001	.065
Emotional problems	2.32 (1.96)	3.08 (2.12)	4.08 (2)	**0.90 [0.49, 1.31]**	**0.38 [0.20, 0.55]**	**0.48 [0.05, 0.90]**	15.66	<.001	.047
Conduct problems	1.86 (1.53)	2.35 (1.62)	2.52 (1.83)	**0.42 [0.02, 0.83]**	**0.31 [0.14, 0.49]**	0.10 [−0.32, 0.52]	7.26	<.001	.021
Hyperactivity	3.44 (2.07)	4.21 (2.23)	4.08 (1.73)	0.31 [−0.09, 0.72]	**0.36 [0.19, 0.54]**	−0.06 [−0.48, 0.36]	8.75	<.001	.025
Peer relationship problems	1.53 (1.49)	2.27 (1.90)	2.71 (2.05)	**0.77 [0.36, 1.19]**	**0.45 [0.28, 0.63]**	0.23 [−0.20, 0.66]	16.24	<.001	.049
Prosocial behavior	8.38 (1.56)	8.24 (1.73)	7.96 (1.97)	−0.27 [−0.67, 0.14]	−0.09 [−0.26, 0.09]	−0.16 [−0.58, 0.26]	1.12	.328	.0004

*Note.* 0–1 ACEs group, *n*= 393; 2–3 ACEs group, *n* = 183; 4 or more ACEs group, *n* = 25. Sample size for each model varies between 595 and 598 depending on the occasional missing data. Extreme values were winsorized to the lower/upper extreme values. Boldface indicates significant group differences where confidence interval does not cross 0. Bonferroni corrected alpha, α = .003.

Regressions were run with dummy coded latent class and cumulative risk variables concurrently for SDQ scales, minus prosocial behavior (see Table 5). At the Bonferroni corrected alpha level, the *community challenges* class significantly contributed to the model for the peer problems scale. The 2 to 3 ACEs cumulative risk group was a significant contributor for total difficulties, emotional symptoms, hyperactivity, and peer problems, while the 4 or more ACEs group was significant for emotional symptoms.

**Table 5.** Associations Between Latent Class and Cumulative Risk Groupings and Outcomes.

Outcome	Community Challenges		Household Challenges		2–3 ACEs		4+ ACEs	
	β	*p*	β	*p*	β	*p*	β	*p*
Total difficulties	.115	.01	−.078	.077	**.239**	<.001	.12	.011
Emotional problems	.060	.189	−.074	.102	.**178**	<.001	.**172**	<.001
Conduct problems	.061	.191	.024	.599	.124	.005	.051	.303
Hyperactivity	.036	.428	−.043	.349	.**172**	<.001	.060	.219
Peer relationship problems	**.181**	<.001	−.116	.008	**.199**	<.001	.010	.034

*Note.* Regressions were not run for the prosocial behavior outcome because neither latent class nor cumulative risk models were significant in the first instance. Boldface indicates significant β value at the Bonferroni corrected α = .003.

## Discussion

This study evaluated two different approaches in operationalizing multiple ACEs in a U.K. household cohort by testing how latent classes and cumulative risk scores of ACEs related to domains of behavioral and emotional problems in childhood. The findings add to the growing literature adopting LCA in the study of ACEs and offers insight into the explanatory value of the person-centered and cumulative risk approaches for behavioral and emotional outcomes in children.

Using LCA, three homogeneous classes were extracted: *low ACEs*, *community challenges*, and *household challenges*. The *low ACEs* class had a low probability of all items, the *community challenges* class had a much larger probability of bullying, adverse neighborhood, and emotional abuse, while the *household challenges* class had a high probability of emotional abuse and physical discipline. The class solution implies that ACEs either co-occurred mostly within the household, or in the wider community. The presence of a class characterized by little or no exposure to ACEs is in line with findings elsewhere, but the absence of a high ACEs class was unexpected. This may be due to the low sample size unable to capture the high ACEs group, or the absence of some ACEs observed in the dataset.

Comparison of different classes found that the *community challenges* class had worse problems, especially for total difficulties, peer problems, and emotional symptoms. The co-occurrence of emotional abuse in addition to adversities in the community might contribute to the potency of this co-occurrence. There is already strong evidence to link bullying victimization to mental health problems (Moore et al., 2017), and a recent meta-analysis found that perceived neighborhood crime was strongly associated with mental health outcomes (Baranyi et al., 2021). The large effects associated with the combination of these adversities is consistent with the literature elsewhere.

It is perhaps counterintuitive that the *household challenges* class scored similarly to a class with low probabilities of all adversities, given well-documented severe effects of child maltreatment (e.g., Gilbert et al., 2009). One potential explanation is that the emotional abuse and physical discipline items were adapted from a parent-report questionnaire about parenting styles (see Robinson et al., 1995) and therefore might be inappropriate to use approximate abusive parenting practices. Alternatively, the questionnaire being parent-reported might have led to underreporting as parents have been found to underreport ACEs compared to their offspring (Fisher et al., 2011).

Comparison of means between cumulative risk groups (0–1 ACEs, 2–3 ACEs, 4 or more ACEs) found that the 2 to 3 ACEs and 4 or more ACEs groups had worse difficulties scores compared to the 0 to 1 ACEs group. Observed differences were larger for internalizing outcomes (emotional symptoms, peer problems) than externalizing problems (hyperactivity, conduct problems), which was also observed in the latent class models. This could indicate that the adversities included in this study are more closely related to internalizing problems than externalizing problems. It has been found elsewhere that certain ACEs (e.g., physical abuse, sexual abuse, and physical neglect) predict externalizing problems better than internalizing problems (Petrenko et al., 2012), which supports the use of person-centered approaches to understand the relationship between co-occurring ACEs and specific outcomes.

Formal comparisons between latent class and cumulative risk models were made by comparing Hay’s ω^2^ values, and by including both latent class and cumulative risk groupings in regression models. Person-centered models explained more variance in the peer problems subscale. However, the cumulative risk model explained more variance for the remaining difficulties. In regression models where dummy variables of both latent class and cumulative risk groupings were included, the *community challenges* group was a significant contributor to the peer problems outcome. The 2 to 3 ACEs group was a significant contributor to the total difficulties, emotional symptoms, hyperactivity, and peer problems, and the 4 or more ACEs group was a significant contributor to emotional symptoms. This suggests that even when accounting for the number of ACEs, the *community challenges* group provides unique insight to explaining peer problems. This further suggests that the person-centered approach may be a useful supplementary method of researching ACEs, specifically for the development of tailored intervention strategies.

These findings should be interpreted in the context of similar comparative studies. Other studies (e.g., Merians et al., 2019) have examined ACEs using retrospective self-report in adulthood, which has been found to produce only modest overlap with prospective self-report in identifying occurrence of abuse (Baldwin et al., 2019). Additionally, young adults retrospectively reporting on ACEs have reported experiencing more ACEs (Radford et al., 2013), so differences in results between our study and previous studies could be due to the confounds such as memory, or the comparison of ACEs experienced up until age 10 years to ACEs experienced up until age 18 years. Indeed, Lacey et al. (2020) found different results based on prospectively and retrospectively reported ACEs in relation to inflammation. Furthermore, our study measured outcomes in childhood, whereas both Merians et al. (2019) and Lacey et al. (2020) measured adult outcomes. It is reasonable to expect different magnitudes of effect between ACEs and outcomes in childhood compared to adulthood, even if outcomes are similar in valence. However, we cannot imply the development or persistence of these problems as our analyses are cross-sectional. Future research designs could benefit from comparing person-centered and cumulative risk approaches across time.

Our findings could be of clinical interest to identifying children at high risk of emotional and behavioral problems in the community. Indeed, one of the advantages of the person-centered approach is the possibility of tailoring intervention strategies to different groups of young people based on the typologies of ACEs (Lanza & Rhoades, 2013). This is particularly useful due to the difficulty of recommending interventions based on a cumulative risk score (Finkelhor, 2018). Future studies should additionally examine whether latent classes of ACEs potentiate specific mediating factors that could be targets for intervention. Theorists have highlighted some possible markers of future difficulties (see McCrory & Viding, 2015). Some research using the cumulative risk score of ACEs has found support for longitudinal mediational pathways (e.g., Iob et al., 2020) that could be novel targets for screening or intervention following exposure to multiple ACEs.

The conclusions drawn in this study must be considered in the context of several limitations. First, class enumeration statistics did not unanimously support one solution in the LCA. This might be explained by the relatively small sample size which produces difficult modeling conditions and compromises the performance of AIC and BIC (Yang, 2006). However, our class solution was theoretically meaningful and demonstrated external validity through associations with relevant outcomes. Second, the ACE items were drawn from a mixture of self-report and parent-report, meaning that our results are vulnerable to underreporting from parents, or common-method variance bias from self-report. It is unclear to what extent these biases impact estimations, but the combination of two types of data collection likely reduces the effect of common-method variance. Third, data regarding important ACEs such as sexual abuse were not observed. However, several ACEs that are usually measured were included, as well as additional items such as bullying victimization. This study fits into the literature examining the operationalization of ACEs, both items to be included and how to model the effect of multiple risks. Finally, it should be noted that the data was collected 9 years ago. It will be important for future studies with more recent data to test whether these relationships endure.

Future studies should consider potential confounders of the relationship between ACEs and psychosocial outcomes, including age of onset, length of exposure, severity, genetic variation, and birth risks in future research (Debowska et al., 2017; Hughes et al., 2017). Other studies have found different latent classes of adversity for boys and girls (Haahr-Pedersen et al., 2020). Based on the observed means, it might be worthwhile to investigate latent classes based on ethnicity and other demographic variables. These could highlight targets for intervention to reduce inequalities, which future studies should continue to explore. Further avenues for future work include the comparison with network analytic approaches (see de Vries et al., 2022; Pollman et al., 2022).

## Conclusion

This research study contributes to the ACEs literature by formulating latent classes in a U.K. sample of children and comparing person-centered and cumulative risk approaches in operationalizing ACEs. Results suggest that the cumulative risk approach accounts for more variance in most regards, but that the person-centered approach generates unique insights. Both cumulative risk and person-centered approaches characterized ACEs well, and specific latent classes conferred risk for specific problems in childhood. Future studies should explore the usefulness of cumulative risk (dichotomized or ordinal) and person-centered approaches, include a broad array of ACEs, and utilize longitudinal data to compare these competing approaches at different stages in childhood and their relevance to adulthood.

## Supplemental Material

sj-docx-1-jiv-10.1177_08862605231153877 – Supplemental material for Comparison of Person-Centered and Cumulative Risk Approaches in Explaining the Relationship Between Adverse Childhood Experiences and Behavioral and Emotional Problems Click here for additional data file. Supplemental material, sj-docx-1-jiv-10.1177_08862605231153877 for Comparison of Person-Centered and Cumulative Risk Approaches in Explaining the Relationship Between Adverse Childhood Experiences and Behavioral and Emotional Problems by George Hales, Agata Debowska, Richard Rowe, Daniel Boduszek and Liat Levita in Journal of Interpersonal Violence

sj-docx-2-jiv-10.1177_08862605231153877 – Supplemental material for Comparison of Person-Centered and Cumulative Risk Approaches in Explaining the Relationship Between Adverse Childhood Experiences and Behavioral and Emotional Problems Click here for additional data file. Supplemental material, sj-docx-2-jiv-10.1177_08862605231153877 for Comparison of Person-Centered and Cumulative Risk Approaches in Explaining the Relationship Between Adverse Childhood Experiences and Behavioral and Emotional Problems by George Hales, Agata Debowska, Richard Rowe, Daniel Boduszek and Liat Levita in Journal of Interpersonal Violence

sj-docx-3-jiv-10.1177_08862605231153877 – Supplemental material for Comparison of Person-Centered and Cumulative Risk Approaches in Explaining the Relationship Between Adverse Childhood Experiences and Behavioral and Emotional Problems Click here for additional data file. Supplemental material, sj-docx-3-jiv-10.1177_08862605231153877 for Comparison of Person-Centered and Cumulative Risk Approaches in Explaining the Relationship Between Adverse Childhood Experiences and Behavioral and Emotional Problems by George Hales, Agata Debowska, Richard Rowe, Daniel Boduszek and Liat Levita in Journal of Interpersonal Violence

## References

[bibr1-08862605231153877] Akaike H. (1974). A new look at the statistical model identification. IEEE Transactions on Automatic Control, 19(6), 716–723. 10.1109/TAC.1974.1100705

[bibr2-08862605231153877] Arminger G. Stein P. Wittenberg J. (1999). Mixtures of conditional mean- and covariance-structure models. Psychometrika, 64(4), 475–494. 10.1007/BF02294568

[bibr3-08862605231153877] Baldwin J. R. Reuben A. Newbury J. B. Danese A. (2019). Agreement between prospective and retrospective measures of childhood maltreatment: A systematic review and meta-analysis. JAMA Psychiatry, 76(6), 584–593. 10.1001/jamapsychiatry.2019.0097 30892562 PMC6551848

[bibr4-08862605231153877] Baranyi G. Di Marco M. H. Russ T. C. Dibben C. Pearce J. (2021). The impact of neighbourhood crime on mental health: A systematic review and meta-analysis. Social Science & Medicine, 282, 114106. 10.1016/j.socscimed.2021.114106 34139480

[bibr5-08862605231153877] Bellis M. A. Hughes K. Ford K. Rodriguez G. R. Sethi D. Passmore J. (2019). Life course health consequences and associated annual costs of adverse childhood experiences across Europe and North America: A systematic review and meta-analysis. The Lancet Public Health, 4(10), e517–e528. 10.1016/S2468-2667(19)30145-8 PMC7098477 31492648

[bibr6-08862605231153877] Cohen J. (1992). A power primer. Psychological Bulletin, 112(1), 155–159. 10.1037/0033-2909.112.1.155 19565683

[bibr7-08862605231153877] Debowska A. Boduszek D. Sherretts N. Willmott D. Jones A. D. (2018). Profiles and behavioral consequences of child abuse among adolescent girls and boys from Barbados and Grenada. Child Abuse & Neglect, 79, 245–258. 10.1016/j.chiabu.2018.02.018 29486347

[bibr8-08862605231153877] Debowska A. Willmott D. Boduszek D. Jones A. D. (2017). What do we know about child abuse and neglect patterns of co-occurrence? A systematic review of profiling studies and recommendations for future research. Child Abuse & Neglect, 70, 100–111. 10.1016/j.chiabu.2017.06.014 28609690

[bibr9-08862605231153877] de Vries T. R. Arends I. Rod N. H. Oldehinkel A. J. Bültmann U . (2022). Proposing network analysis for early life adversity: An application of life event data. Social Science & Medicine, 296, 114784. 10.1016/j.socscimed.2022.114784 35152049

[bibr10-08862605231153877] Edwards V. J. Holden G. W. Feliiti V. J. Anda R. F. (2003). Relationship between multiple forms of childhood maltreatment and adult mental health in community respondents: Results from the adverse childhood experiences study. American Journal of Psychiatry, 160, 1453–1460. 10.1176/appi.ajp.160.8.1453 12900308

[bibr11-08862605231153877] Felitti V. J. Anda R. F. Nordenberg D. Williamson D. F. Spitz A. M. Edwards V. Koss M. P. Marks J. S. (1998). Relationship of childhood abuse and household dysfunction to many of the leading causes of death in adults: The Adverse Childhood Experiences (ACE) study. American Journal of Preventive Medicine, 14(4), 245–258. 10.1016/S0749-3797(98)00017-8 9635069

[bibr12-08862605231153877] Finkelhor D. (2018). Screening for adverse childhood experiences (ACEs): Cautions and suggestions. Child Abuse & Neglect, 85, 174–179. 10.1016/j.chiabu.2017.07.016 28784309

[bibr13-08862605231153877] Finkelhor D. Shattuck A. Turner H. Hamby S. (2015). A revised inventory of adverse childhood experiences. Child Abuse & Neglect, 48, 13–21. 10.1016/j.chiabu.2015.07.011 26259971

[bibr14-08862605231153877] Fisher H. L. Bunn A. Jacobs C. Moran P. Bifulco A. (2011). Concordance between mother and offspring retrospective reports of childhood adversity. Child Abuse & Neglect, 35, 117–122. 10.1016/j.chiabu.2010.10.003 21354622 PMC3272365

[bibr15-08862605231153877] Gilbert R. Widom C. S. Browne K. Fergusson D. Webb E. Janson S. (2009). Burden and consequences of child maltreatment in high-income countries. Lancet, 373, 68–81. 10.1016/S0140-6736(08)61706-7 19056114

[bibr16-08862605231153877] Goldberg D. P. Oldehinkel T. Ormel J. (1998). Why GHQ threshold varies from one place to another. Psychological Medicine, 28, 915–921. 10.1017/S0033291798006874 9723146

[bibr17-08862605231153877] Goldberg D. P. Williams P. (1988). A users’ guide to the General Health Questionnaire. London: GL Assessment.

[bibr18-08862605231153877] Goodman A. Goodman R. (2009). Strengths and Difficulties Questionnaire as a dimensional measure of child mental health. Journal of American Academy of Child and Adolescent Psychiatry, 48(4), 400–403. 10.1097/CHI.0b013e3181985068 19242383

[bibr19-08862605231153877] Goodman R. Ford T. Simmons H. Gatward R. Meltzer H. (2000). Using the Strengths and Difficulties Questionnaire (SDQ) to screen for child psychiatric disorders in a community sample. British Journal of Psychiatry, 177(6), 534–539. 10.1192/bjp.177.6.534 11102329

[bibr20-08862605231153877] Haahr-Pedersen I. Perera C. Hyland P. Vallières F. Murphy D. Hansen M. Spitz P. Hansen P. Cloitre M. (2020). Females have more complex patterns of childhood adversity: Implications for mental, social, and emotional outcomes in adulthood. European Journal of Psychotraumatology, 11(1), 1708618. 10.1080/20008198.2019.1708618 32002142 PMC6968572

[bibr21-08862605231153877] Hales G. Saribaz Z. E. Debowska A. Rowe R. (2022). Links of adversity in childhood with mental and physical health outcomes: A systematic review of longitudinal mediating and moderating mechanisms. Trauma, Violence, & Abuse. Advanced online publication. 10.1177/15248380221075087 PMC10240645 35226575

[bibr22-08862605231153877] Hughes K. Bellis M. A. Hardcastle K. A. Sethi D. Butchart A. Mikton C. Jones L. Dunne M. P. (2017). The effect of multiple adverse childhood experiences on health: A systematic review and meta-analysis. Lancet Public Health, 2, e356–e366. 10.1016/S2468-2667(17)30118-4 29253477

[bibr23-08862605231153877] Hughes K. Ford K. Bellis M. A. Glendinning F. Harrison E. Passmore J. (2021). Health and financial costs of adverse childhood experiences in 28 European countries: A systematic review and meta-analysis. Lancet Public Health, 6(11), e848–e857. 10.1016/S2468-2667(21)00232-2 PMC8573710 34756168

[bibr24-08862605231153877] Iob E. Lacey R. Steptoe A. (2020). Adverse childhood experiences and depressive symptoms in later life: Longitudinal mediation effects of inflammation. Brain, Behavior, and Immunity, 90, 97–107. 10.1016/j.bbi.2020.07.045 32755647

[bibr25-08862605231153877] Kirk R. E. (1996). Practical significance: A concept whose time has come. Educational and Psychological Measurement, 56(5), 746–759. 10.1177/0013164496056005002

[bibr26-08862605231153877] Lacey R. E. Pereira S. M. P. Li L. Danese A. (2020). Adverse childhood experiences and adult inflammation: Single adversity, cumulative risk and latent class approaches. Brain, Behavior, and Immunity, 87, 820–830. 10.1016/j.bbi.2020.03.017 32201253 PMC7327510

[bibr27-08862605231153877] Lanier P. Maguire-Jack K. Lombardi B. Frey J. Rose R. A. (2018). Adverse childhood experiences and child health outcomes: Comparing cumulative risk and latent class approaches. Maternal and Child Health Journal, 22(3), 288–297. 10.1007/s10995-017-2365-1 28929420

[bibr28-08862605231153877] Lanza S. T. Rhoades B. L. (2013). Latent class analysis: An alternative perspective on subgroup analysis in prevention and treatment. Prevention Science, 14(2), 157–168. 10.1007/s11121-011-0201-1 21318625 PMC3173585

[bibr29-08862605231153877] Lo Y. Mendell N. R. Rubin D. B. (2001). Testing the number of components in a normal mixture. Biometrika, 88(3), 767–778. 10.1093/biomet/88.3.767

[bibr30-08862605231153877] McCrory E. J. Viding E. (2015). The theory of latent vulnerability: Reconceptualizing the link between childhood maltreatment and psychiatric disorder. Development and Psychopathology, 27, 493–505. 10.1017/S0954579415000115 25997767

[bibr31-08862605231153877] Merians A. N. Baker M. R. Frazier P. Lust K. (2019). Outcomes related to adverse childhood experiences in college students: Comparing latent class analysis and cumulative risk. Child Abuse & Neglect, 87, 51–64. 10.1016/j.chiabu.2018.07.020 30064695

[bibr32-08862605231153877] Moore S. E. Norman R. E. Suetani S. Thomas H. J. Sly P. D. Scott J. G. (2017). Consequences of bullying victimization in childhood and adolescence: A systematic review and meta-analysis. World Journal of Psychiatry, 7(1), 60–76. 10.5498/wjp.v7.i1.60 28401049 PMC5371173

[bibr33-08862605231153877] Muthén L. K. Muthén B. O. (1998–2017). Mplus user’s guide (8th ed.). Muthén & Muthén.

[bibr34-08862605231153877] Nylund K. L. Asparouhov T. Muthén B. O. (2007). Deciding on the number of classes in latent class analysis and growth mixture modelling: A Monte Carlo simulation study. Structural Equation Modeling, 14(4), 535–569. 10.1080/10705510701575396

[bibr35-08862605231153877] ONS. (2012). Ethnicity and national identity in England and Wales: 2011. Office for National Statistics. Retrieved March 21, 2022, from https://www.ons.gov.uk/peoplepopulationandcommunity/culturalidentity/ethnicity/articles/ethnicityandnationalidentityinenglandandwales/2012-12-11

[bibr36-08862605231153877] Petrenko C. L. Friend A. Garrido E. F. Taussig H. N. Culhane S. E. (2012). Does subtype matter? Assessing the effects of maltreatment on functioning in preadolescent youth in out-of-home care. Child Abuse & Neglect, 36(9), 633–644. 10.1016/j.chiabu.2012.07.001 22947490 PMC3445713

[bibr37-08862605231153877] Pollman A. Fritz J. Barker E. Fuhrmann D. (2022). Networks of adversity in childhood and adolescence and their relationship to adult mental health. Research on Child and Adolescent Psychopathology. Advanced online publication. 10.1007/s10802-022-00976-4 PMC10661796 36331717

[bibr38-08862605231153877] Radford L. Corral S. Bradley C. Fisher H. L. (2013). The prevalence and impact of child maltreatment and other types of victimization in the UK: Findings from a population survey of caregivers, children and young people and young adults. Child Abuse & Neglect, 37, 801–813. 10.1016/j.chiabu.2013.02.004 23522961

[bibr39-08862605231153877] Ramaswamy V. Desarbo W. S. Reibstein D. J. Robinson W. T. (1993). An empirical pooling approach for estimating marketing mix elasticities with PIMS data. Marketing Science, 12(1), 103–124. 10.1287/mksc.12.1.103

[bibr40-08862605231153877] Robinson C. C. Mandleco B. Olsen S. F. Hart C. H. (1995). Authoritative, authoritarian, and permissive parenting practices: Development of a new measure. Psychological Reports, 77, 819–830. 10.2466/pr0.1995.77.3.819

[bibr41-08862605231153877] Schwarz G. (1978). Estimating the dimension of a model. The Annals of Statistics, 6(2), 461–464.

[bibr42-08862605231153877] Sclove S. L. (1987). Application of model-selection criteria to some problems in multivariate analysis. Psychometrika, 52(3), 333–343. 10.1007/BF02294360

[bibr43-08862605231153877] Sharratt K. Boduszek D. Gallagher B. Jones A. (2018). Factor structure and factorial invariance of the Strengths and Difficulties Questionnaire among children of prisoners and their parents. Child Indicators Research, 11(2), 649–660. 10.1007/s12187-017-9464-9 29527244 PMC5838117

[bibr44-08862605231153877] StataCorp. (2019). Stata Statistical Software: Release 16. College Station, TX: StataCorp LLC.

[bibr45-08862605231153877] University of Essex. (2020). Understanding society: Waves 1–10, 2009–2019 and harmonised BHPS: Waves 1–18, 1991–2009 [data collection] (13th ed.). UK Data Service.

[bibr46-08862605231153877] Yang C. (2006). Evaluating latent class analysis models in qualitative phenotype identification. Computational Statistics & Data Analysis, 50, 1090–1104. 10.1016/j.csda.2004.11.004

