# Supplementary material for: Comparison of Person-Centered and Cumulative Risk Approaches in Explaining the Relationship Between Adverse Childhood Experiences and Behavioral and Emotional Problems
Source: J Interpers Violence. 2023 Feb 10;38(13-14):8065–87. doi: 10.1177/08862605231153877 (PMC10326363; doi:10.1177/08862605231153877)
Supplement: sj-docx-1-jiv-10.1177_08862605231153877 – Supplemental material for Comparison of Person-Centered and Cumulative Risk Approaches in Explaining the Relationship Between Adverse Childhood Experiences and Behavioral and Emotional Problems [file sj-docx-1-jiv-10.1177_08862605231153877.docx]

**Supplementary Table 1**

*Adversities and their contributing items.*

| Adversities | Items |
| --- | --- |
| Physical discipline^a^ | “I use physical punishment as a way of disciplining [child]” |
|  | “I spank [child] when [he/she] is disobedient” |
|  | “I explode in anger towards [child]” |
|  | “I grab [child] when [he/she] is being disobedient” |
|  | “I slap [child] when [he/she] misbehaves” |
| Emotional abuse^a^ | “I scold and criticise to make [child] improve” |
|  | “I scold or criticise when [child]'s behaviour doesn't meet my expectations” |
| Supervisory Neglect^a^ | “I punish [child] by putting [him/her] somewhere alone with little or no explanation” |
| Maternal Psychological Distress^a^ | Reported using the Short General Health Questionnaire (GHQ-12; Goldberg & Williams, 1988) |
| Educational disinterest^b^ | “My parents are interested in how I do at school” |
|  | “My parents come to school parents’ evenings” |
| Bullying Victimisation^b^ | “How often do you get physically bullied at school, for example getting  hit, pushed around or threatened, or having belongings stolen?” |
|  | “How often do you get bullied in other ways at school such as getting  called names, getting left out of games, or having nasty stories spread  about you on purpose?” |
| Adverse Neighbourhood^b^ | “How much do you worry that you might be a victim of a crime?” |
|  | “How safe would you feel walking alone in this area after dark?” |

*Note*. ^a^ = parent-reported items, ^b^ = child-reported items.
