# Supplementary material for: Comparison of Person-Centered and Cumulative Risk Approaches in Explaining the Relationship Between Adverse Childhood Experiences and Behavioral and Emotional Problems
Source: J Interpers Violence. 2023 Feb 10;38(13-14):8065–87. doi: 10.1177/08862605231153877 (PMC10326363; doi:10.1177/08862605231153877)
Supplement: sj-docx-2-jiv-10.1177_08862605231153877 – Supplemental material for Comparison of Person-Centered and Cumulative Risk Approaches in Explaining the Relationship Between Adverse Childhood Experiences and Behavioral and Emotional Problems [file sj-docx-2-jiv-10.1177_08862605231153877.docx]

**Supplementary Table 2.**

*Latent class and SDQ subscale ANOVAs re-run with sex and ethnicity in the model.*

| Outcome | Model |  | Latent Class | | Sex |  | Ethnicity | |
| --- | --- | --- | --- | --- | --- | --- | --- | --- |
|  | *F* | *p* | *F* | *p* | *F* | *p* | *F* | *p* |
| Total difficulties | 10.51 | <.001 | 11.36 | <.001 | 15.62 | <.001 | 1.01 | .316 |
| Emotional problems | 4.72 | .001 | 7.63 | .001 | 4.22 | .04 | .09 | .76 |
| Conduct problems | 7.15 | <.001 | 3.49 | .031 | 18.55 | <.001 | .95 | .331 |
| Hyperactivity | 11.84 | <.001 | 1.77 | .171 | 37.16 | <.001 | 4.05 | .045 |
| Peer relationship problems | 13.73 | <.001 | 19.76 | <.001 | 12.75 | <.001 | 0 | .975 |
| Prosocial behaviour | 7.37 | <.001 | .52 | .595 | 27.17 | <.001 | .02 | .901 |
