# Supplementary material for: Comparison of Person-Centered and Cumulative Risk Approaches in Explaining the Relationship Between Adverse Childhood Experiences and Behavioral and Emotional Problems
Source: J Interpers Violence. 2023 Feb 10;38(13-14):8065–87. doi: 10.1177/08862605231153877 (PMC10326363; doi:10.1177/08862605231153877)
Supplement: sj-docx-3-jiv-10.1177_08862605231153877 – Supplemental material for Comparison of Person-Centered and Cumulative Risk Approaches in Explaining the Relationship Between Adverse Childhood Experiences and Behavioral and Emotional Problems [file sj-docx-3-jiv-10.1177_08862605231153877.docx]

**Supplementary Table 3.**

*Cumulative risk and SDQ subscale ANOVAs re-run with sex and ethnicity in the model.*

| Outcome | Model |  | Cumulative risk | | Sex |  | Ethnicity | |
| --- | --- | --- | --- | --- | --- | --- | --- | --- |
|  | *F* | *p* | *F* | *p* | *F* | *p* | *F* | *p* |
| Total difficulties | 15.76 | <.001 | 21.54 | <.001 | 15.75 | <.001 | 2.24 | .135 |
| Emotional problems | 9.04 | <.001 | 16.21 | <.001 | 4.67 | .031 | .04 | .837 |
| Conduct problems | 8.88 | <.001 | 6.82 | .001 | 18.95 | <.001 | 1.12 | .29 |
| Hyperactivity | 15.56 | <.001 | 8.71 | <.001 | 36.78 | <.001 | 5.37 | .021 |
| Peer relationship problems | 11.52 | <.001 | 15.43 | <.001 | 12.54 | <.001 | .25 | .621 |
| Prosocial behaviour | 7.47 | <.001 | .73 | .484 | 27.53 | <.001 | 0 | .956 |
